# Antibiotics for Rheumatologic Diseases: A Critical Review

**DOI:** 10.3390/ijms262110527

**Published:** 2025-10-29

**Authors:** Matthew E. Falagas, Panagiotis Stathopoulos, Dimitrios S. Kontogiannis, Iva D. Tzvetanova

**Affiliations:** 1 Alfa Institute of Biomedical Sciences, 9 Neapoleos Street, Marousi, 151 23 Athens, Greece; 2 School of Medicine, European University Cyprus, 2404 Nicosia, Cyprus; i.tzvetanova@euc.ac.cy; 3 Department of Medicine, Tufts University School of Medicine, Boston, MA 02111, USA

**Keywords:** antibiotics, rheumatologic diseases, immunomodulation, autoimmunity, infection, reactive arthritis, rheumatoid arthritis, lyme disease, whipple’s disease, rheumatic fever

## Abstract

Antibiotics have been traditionally used to treat patients with infectious diseases. However, recent investigations have highlighted their immunomodulating features. Additionally, they have been used to treat patients with rheumatologic diseases of proven infectious etiology. Thus, an emerging body of literature is developing on the potential role of antibiotics in managing patients with rheumatologic diseases, which are primarily characterized by autoimmune-driven inflammation. We critically review the potential use of antibiotics in rheumatology, focusing on both their direct antimicrobial actions and immunomodulatory effects. We also examine the potential clinical applications, underlying pharmacological mechanisms, controversies, and future research directions. Databases of biomedical research (PubMed, Scopus, Web of Science, and Cochrane) and Google Scholar were searched. The critical evaluation of the available data suggests that antibiotics should be used only for patients with rheumatologic diseases with a clear infectious etiology. These indications are the treatment and prevention of recurrence of rheumatic fever, Whipple’s disease, and early Lyme disease. Additionally, antibiotics may be considered for early administration in patients with reactive arthritis. Until data from robust clinical trials support the consideration of antibiotics in other rheumatologic diseases, beyond those with a clear infectious etiology, clinicians should follow the internationally relevant guidelines and avoid their use in treating such diseases in this patient population. Further studies may offer additional data for using antibiotics in treating patients with additional rheumatologic diseases, especially in cases where conventional treatments have inadequate effectiveness or are associated with considerable adverse events.

## 1. Introduction

Rheumatologic diseases are common, affecting a significant proportion of the world’s population and contributing substantially to morbidity, mortality, and economic burden [1]. They include a wide range of disorders that primarily affect the musculoskeletal system and connective tissue [2]. These conditions can be either localized or systemic in nature, and their impact varies widely, ranging from mild discomfort to debilitating disability [3]. Some rheumatologic diseases, such as osteoarthritis and gout, primarily involve the joints, presenting with pain, stiffness, and swelling [4]. In contrast, other conditions, including rheumatoid arthritis (RA) and systemic lupus erythematosus (SLE), are systemic diseases that cause multisystem involvement, leading to a wide range of clinical manifestations and a broad spectrum of clinical phenotypes. Notably, these disease phenotypes are often indistinct, with each patient presenting with a unique constellation of clinical signs and symptoms, as well as laboratory and imaging abnormalities [5,6].

The pathophysiology of rheumatologic diseases involves chronic inflammation and autoimmune dysregulation. This applies to some of the most common relevant diseases, including rheumatoid arthritis, spondyloarthropathies, and systemic lupus erythematosus [7]. The current understanding of their pathogenesis supports a mixture of effects of genetic predisposition, infectious agents, and environmental triggers [8,9]. The evolving clarification of the immunomodulatory effects of the antibiotics, including their impact on the human microbiota, suggests a potential role for these drugs in the treatment of patients with rheumatologic diseases [10].

In addition, there are specific rheumatologic diseases in which chronic infection is a basic mechanism leading to chronic inflammation, resulting in subsequent clinical manifestations. Examples of such diseases are Whipple’s disease and Lyme arthritis, in which antibiotics may have a special therapeutic position [11,12,13].

These observations suggest the potential role of antibiotics in the treatment of patients with rheumatologic diseases [14,15]. This may be accomplished via their direct antimicrobial activity, which leads to the elimination of microorganisms associated with the pathogenesis of some rheumatologic diseases, and via their immunomodulatory effects, which may modify the progression of rheumatologic diseases [16].

In this article, we critically examine some aspects of the intersection of infectious diseases and rheumatology by focusing on data on the therapeutic role of antibiotics in patients with rheumatologic diseases. We include diseases in which our current understanding of their pathophysiology supports that infection is the basic etiologic factor, acts as an immunologic trigger, or mimics autoimmunity.

We did not include in this article other essential aspects of the intersection of infectious diseases and rheumatology, specifically the use of antimicrobial agents for the prevention and treatment of infections in patients with rheumatologic diseases, given their increased susceptibility to infections due to the immunosuppressive properties of several relevant therapies [17,18,19,20,21]. Also, we did not examine data on vaccines for preventing infections in such patients. In addition, we did not include data on the role of anti-malarial type medications, such as hydroxychloroquine, which have specific indications for patients with various rheumatologic diseases, such as discoid lupus erythematosus. Furthermore, we did not review the absolute indication for the use of antibiotics in patients with septic arthritis, including gonococcal arthritis, given the direct pathogenicity of microorganisms in the affected joint [22]. Also, we did not examine findings on the role of infectious agents such as the hepatitis B virus and *Mycobacterium tuberculosis*, which may contribute to the pathogenesis of rheumatologic diseases. However, their presence poses concerns against the use of immunosuppressive treatments, unless they are controlled effectively [23,24].

In addition, we did not include data on various clinical aspects of rheumatologic diseases that may be interpreted as consequences of infectious diseases in this article. For example, various rheumatologic diseases may manifest with symptoms and signs strongly suggesting infectious cellulitis [25]. Another example is cases of granulomatosis with polyangiitis (GPA), previously called Wegener’s disease, presenting as a pulmonary infection [26]. On the other hand, several antimicrobial agents may cause adverse events, suggesting various manifestations of a rheumatologic disease, such as serum sickness-like hypersensitivity allergic reactions after using beta-lactam antibiotics [27].

This review was conducted as a narrative synthesis of the available literature. Searches were performed in PubMed and Scopus through July 2025 using the terms “antibiotics,” “immunomodulation,” and “rheumatologic diseases.” Peer-reviewed articles, clinical trials, and key experimental studies were included without language restriction, with a focus on evidence relevant to both antimicrobial and immune-mediated mechanisms. The objective was to critically summarize the biological and clinical intersections between infectious diseases and rheumatology.

## 2. Infection and Autoimmunity

Clinicians have observed the onset or exacerbation of rheumatologic diseases after infections. Classic examples are reactive arthritis, an asymmetric oligoarthritis typically following genitourinary or gastrointestinal infection, and rheumatic fever, a post-streptococcal autoimmune inflammatory disease affecting joints, heart, and skin. Reactive arthritis may follow infections of the genitourinary system due to *Chlamydia trachomatis* or infections of the gastrointestinal tract due to *Yersinia* spp., *Shigella* spp., and *Salmonella* spp. [28,29,30,31,32]. Also, rheumatic fever may follow pharyngeal infection caused by *Streptococcus pyogenes*.

Several possible mechanisms underlie the interface between infection and autoimmunity. Antigens of microorganisms may resemble molecules of humans (self-proteins) and may initiate and continue an autoimmunity reaction. This mechanism is called molecular mimicry, in which shared epitopes between microbial and host antigens lead to cross-reactivity and subsequently trigger an autoimmune response against self-tissues [33,34,35]. Also, the immune response directed against a microbial agent may expand to include self-antigens released during tissue damage, a phenomenon known as epitope spreading [36,37]. Bystander activation refers to the release of inflammatory cytokines during infection, which may activate autoreactive lymphocytes that would otherwise remain quiescent [38]. In addition, chronic active infection may lead to chronic inflammation and autoimmunity, due to persistent stimulation of the immune system [39]. Table 1 summarizes the basic pathogenic mechanisms of infections that lead to rheumatologic diseases.

Basic clinical and laboratory observations that support the interface between infection and autoimmunity include the temporal association between infection and onset of a rheumatologic disease, the identification of cross-reactive antibodies against microbial antigens and self-antigens, and the documentation of microbial genetic molecules (nucleic acids) in inflamed tissues [40,41]. Subsequently, antibiotics may have a role in patients with rheumatologic diseases by curing active infections and potentially intervening at the interface of infection and autoimmunity.

Additionally, antibiotics used to treat various infections may lead to alterations in the host microbiota. This effect has also been linked to the development of rheumatic diseases, such as seronegative spondyloarthropathies, a group of inflammatory rheumatic disorders that primarily affect the spine and entheses and lack rheumatoid factor positivity, as well as diseases belonging to the broader spectrum of autoimmune diseases [42,43].

## 3. Immunomodulatory Effects of Antibiotics

The immunomodulatory effects of antibiotics have been extensively studied, and numerous hypotheses have been proposed regarding the mechanisms by which they exert immunomodulatory effects [44]. It is important to distinguish between the immunologic effects that arise indirectly from infection control and those that result directly from antibiotic exposure. By eliminating or suppressing pathogens, antibiotics can indirectly modulate immune activity through reduced antigenic stimulation and changes in the host microbiota. In contrast, several antibiotic classes also exhibit direct immunomodulatory properties, acting on host immune cells and signaling pathways independently of their antimicrobial effects.

First, it is well established that infections themselves are immunomodulatory events, and reducing the burden of an infection with antibiotic treatment may alter the immune response, though not necessarily reduce it. For example, the eradication of a spirochetal infection, such as syphilis, leptospirosis, or borelliosis, can trigger the Jarisch-Herxheimer reaction, where bacterial cell lysis releases antigenic molecules and endotoxin-like substances, which may provoke an inflammatory response [45]. The effects on host intestinal flora, which play a significant role in immune system physiology, as well as the reduction in virulence factors in pathogenic bacteria, are additional ways in which antibiotics exert immunomodulatory effects. However, all of these mechanisms are indirect, and the hypothesis that antibiotics may directly exert immunomodulatory actions is gaining increasing attention. Additionally, a direct immunomodulatory action of antibiotics has been proposed. Relevant studies have shown that immune cells behave differently when exposed to certain antibiotics, with a modified response after exposure to inflammatory agents [44].

Evolving experimental data suggest that several classes of antibiotics have immunomodulatory features that may modify the course of rheumatologic diseases and other diseases [46,47,48,49]. These features involve modulation of cytokines, including inhibition of pro-inflammatory enzymes, and suppression of immune cell activation [50,51]. Experimental data on the immunomodulatory effects of selected antibiotics, especially those considered for clinical use in patients with rheumatologic diseases, are reviewed below. Table 2 summarizes the main mechanisms of the immunomodulatory effects of these antibiotics.

### 3.1. Macrolides

Azithromycin and clarithromycin, macrolide antibiotics, have proven immunomodulatory actions, including anti-inflammatory properties [52,53]. They reduce the production of pro-inflammatory cytokines such as IL-1β, IL-6, and TNF-α by inhibiting the transcription of the relevant genes encoding these proteins [54]. They also have other immunomodulatory actions, including the stabilization of lysosomal membranes, the suppression of the oxidative burst, and the reduction in neutrophil chemotaxis and adhesion [55].

These features have led investigators to use macrolide antibiotics in patients with chronic inflammatory airway diseases, such as diffuse pan-bronchiolitis [56]. However, the clinical data on the use of these antibiotics in patients with rheumatologic diseases is limited, despite macrolides having been shown to suppress the expression of inflammatory markers and, most importantly, decrease the severity of arthritis in exploratory experimental models. In experimental in vivo studies, particularly in collagen-induced arthritis models, azithromycin has been shown to markedly reduce disease severity and inflammatory cytokine expression through modulation of unfolded protein response pathways, including inhibition of GRP78 [57].

### 3.2. Tetracyclines

Minocycline, doxycycline, and omadacycline are tetracycline antibiotics with immunomodulatory actions [58]. They include the inhibition of matrix metalloproteinases (MMPs), which are associated with cartilage and joint tissue degradation in patients with RA [59]. Additionally, they reduce T-lymphocyte activation and proliferation, leading to the suppression of the production of pro-inflammatory cytokines [60]. In addition, they inhibit the activation of macrophages and microglial cells.

Clinical trials have shown that tetracyclines, particularly minocycline, can improve joint symptoms and laboratory markers of inflammation in rheumatoid arthritis. A 26-week double-blind study reported significant reductions in laboratory indices of disease activity, although clinical improvements were modest [61]. The larger 48-week multicenter trial (named MIRA) demonstrated significant improvement in joint tenderness, swelling, and inflammatory markers, confirming both safety and moderate efficacy in mild-to-moderate RA [62]. Similarly, another study found that 65% of patients with early seropositive RA achieved ≥50% symptom improvement compared with 13% in the placebo group [63]. Overall, these findings indicate that tetracyclines may offer modest benefit in early disease, while effects in advanced RA remain limited, likely due to irreversible joint damage. Also, adverse events, particularly from the skin, such as photosensitivity and skin pigmentation, pose concerns for their chronic use in patients with rheumatologic diseases.

### 3.3. Fluoroquinolones

Fluoroquinolones, such as ciprofloxacin and levofloxacin, can have anti-inflammatory actions by reducing the production of pro-inflammatory cytokines such as TNF-α, IL-1β, and IL-6, and by inhibiting the activation of a key transcription factor involved in inflammatory responses, nuclear factor-kappa B (NF-κB) [64,65]. They can also have immunomodulatory effects by interfering with the function of various cells, including neutrophils (suppression of the neutrophil chemotaxis and oxidative burst), macrophages, and lymphocytes (reduction in antibody production and T-cell proliferation) [66]. Also, fluoroquinolones may induce apoptosis in immune cells [67].

However, these interesting experimental observations have not been translated into clinical data on the use of fluoroquinolones for patients with rheumatologic diseases. In addition, there are substantial concerns against the use of fluoroquinolones in the treatment of rheumatologic diseases. They include the increased risk of adverse events of these antibiotics from the tendons in patients with underlying rheumatologic diseases [68]. In addition, the rarely seen neuropathy as an adverse event of fluoroquinolones is a concern for this patient population [69]. Also, these antibiotics may cause drug-induced lupus [70].

### 3.4. Sulfasalazine

Sulfasalazine is a drug that contains sulfapyridine (a sulfonamide substance) and 5-aminosalicylic acid. It has considerable immunomodulatory actions, including changes in the function of B-lymphocytes, suppression of the production of IL-1 and TNF, and inhibition of NF-κB [71]. It has been used in patients with RA and seronegative spondyloarthropathies with noticeable results. The contribution of its direct antimicrobial activity to its beneficial effect noted in patients with RA is unclear. Also, an interesting area for further investigation is the therapeutic effect of the drug on the microbial gut microflora.

### 3.5. Other Antibiotics

In vitro studies have investigated the effects of various antibiotics on cells of the innate immune system. Piperacillin, an antipseudomonal penicillin, has been shown to inhibit the phagocytic activity of peripheral blood monocytes in vitro [72]. It also increases the levels of pro-inflammatory cytokines, including IL-1β and IL-6, and upregulates the expression of Toll-like receptors (TLRs), which are transmembrane proteins that bind to pathogen-associated molecular patterns (PAMPs) [72].

Linezolid, an oxazolidinone antibiotic often used to treat methicillin-resistant *Staphylococcus aureus* (MRSA) and other Gram-positive infections, has been shown to upregulate the expression of TLRs and inhibit the phagocytic activity of monocytes in sepsis-like conditions in vitro [73]. Through its immunomodulatory effects, it reduces the inflammatory damage induced by pro-inflammatory cytokines, not only in infections but also in general inflammatory conditions [74]. The use of linezolid and its impact on rheumatologic diseases has not been shown yet.

Vancomycin, a glycopeptide with broad-spectrum Gram-positive coverage including MRSA, promotes monocyte phagocytosis, as well as the expression of TLRs in an in vitro sepsis model [73]. Oral vancomycin has been shown to exert immunomodulatory effects in vivo, normalizing laboratory abnormalities and improving clinical symptoms, pathology, and imaging studies in pediatric patients with inflammatory bowel disease (IBD) and primary sclerosing cholangitis (PSC). The effect of vancomycin on rheumatologic disorders has not been studied.

Daptomycin, a lipopeptide with anti-MRSA activity, downregulates TLR expression of peripheral blood mononuclear cells in an in vitro sepsis model [73]. Daptomycin has been shown to inhibit the secretion of pro-inflammatory cytokines and reduce cell inflammation in in vitro cytological studies. Arthritis symptoms were improved in mice with collagen-induced arthritis when administered daptomycin, and the serum levels of IL-1β, TNF-α, and IL-6 were decreased [75]. However, these effects have not been reported in human studies.

Polymyxins, which are last resort antibiotics reserved for treating multidrug-resistant Gram-negative infections, have been reported to have an immunomodulatory action in in vivo experimental studies [76]. Specifically, colistin was found to preserve a protein kinase pathway essential for innate immunity, providing protection from Gram-negative pathogens and potentially inducing host tolerance to these infections [77]. Notably, this action enhances immune response, unlike most of the other antibiotics. While this immunostimulatory activity can enhance antimicrobial defense, it contrasts with the immunosuppressive or immunomodulatory approaches typically desired in rheumatologic disease management, where excessive immune activation drives pathology. Consequently, polymyxins are unlikely to represent viable candidates for disease modification in autoimmune or inflammatory rheumatic disorders.

## 4. Clinical Applications in Rheumatologic Diseases

The following section summarizes clinical contexts where antibiotics are used in rheumatologic diseases, addressing both their antimicrobial activity against causative pathogens and their secondary immunomodulatory effects modifying disease progression.

### 4.1. Lyme Disease and Arthritis

Lyme disease is a multi-systemic disease caused by *Borrelia burgdorferi*, a Gram-negative bacterium. If left untreated, the infection may lead to chronic arthritis. Antibiotic choices for the treatment of patients with *Borrelia burgdorferi* infection include doxycycline, amoxicillin, or ceftriaxone [78]. In cases with continuing joint inflammation despite the appropriate treatment of the infection, immunomodulatory therapy may be considered [79]. However, clinicians should avoid using antibiotics after the initial treatment of *Borrelia burgdorferi* infection, because the pathogenic mechanism at this stage of Lyme disease is sequelae from infection-triggered autoimmunity, not active infection per se [80].

### 4.2. Whipple’s Disease

*Tropheryma whipplei*, a Gram-negative bacterium, is the causative agent of Whipple’s disease, which may cause clinical manifestations from various systems, including the gastrointestinal tract (diarrhea and weight loss) and the joints (arthralgia and, in some patients, arthritis). The identification of genetic molecules of the bacterium by PCR or the bacterium itself by periodic acid-Schiff (PAS) staining of biopsy specimens confirms the diagnosis.

Antibiotic treatment with intravenous ceftriaxone or penicillin G is recommended to initially treat the *Tropheryma whipplei* infection, which usually leads to significant improvement in symptoms [81]. Subsequently, maintenance therapy with oral antibiotics, typically trimethoprim-sulfamethoxazole, is administered for 1–2 years in patients with Whipple’s disease [82].

### 4.3. Rheumatic Fever

Untreated infection of the pharynx by group A Streptococcus can lead to rheumatic fever, a disease characterized by clinically significant manifestations, including fever, arthritis, carditis, skin rash, subcutaneous nodules, and chorea. Rheumatic fever is an autoimmune sequela of this specific infection, especially in children and adolescents.

Antimicrobial treatment (preferably with penicillin in the absence of a history of allergy) is necessary for the treatment of patients with acute streptococcal pharyngitis [83,84,85]. In addition, long-term secondary prevention with penicillin prophylaxis treatment is needed to reduce the probability of recurrence of rheumatic fever and prevent progression to rheumatic heart disease [83,86].

### 4.4. Spondyloarthropathies

Reactive arthritis, psoriatic arthritis, and ankylosing spondylitis are the main spondyloarthropathies. From those diseases, reactive arthritis is frequently preceded by a urogenital or gastrointestinal infection [87]. Early treatment of patients with reactive arthritis with antibiotics such as ciprofloxacin, doxycycline, and azithromycin may be beneficial; however, relevant studies have shown variable outcomes [15,88,89,90]. The effectiveness of antibiotics decreases in cases of chronic reactive arthritis. However, a symptomatic relief was noted in a subset of patients with reactive arthritis who received antibiotics for a prolonged time (up to 6 months).

Gut dysbiosis has been implicated as a contributing factor driving both the pathogenesis as well as the exacerbations of rheumatic diseases, including spondyloarthropathies [91,92]. Notably, data on the pathogenesis of ankylosing spondylitis suggest that disease development may be driven by the presence of specific bacteria, such as *Klebsiella* spp., in the gut of genetically predisposed individuals (e.g., those with positive HLA-B27), as various studies have demonstrated. Still, this hypothesis has not been conclusively established [93]. Additionally, data, although contradictory, exist regarding the presence of *Klebsiella pneumoniae* antibodies associated with intestinal inflammation in patients with ankylosing spondylitis [94,95,96]. This suggests that antibiotics altering microflora could either induce or prevent dysbiosis, and might even have a beneficial effect on patients with ongoing autoinflammatory disease [42,43]. Antibiotics-induced microflora alteration and an impact on ankylosing spondylitis is supported by data on the beneficial use of moxifloxacin, rifaximin, as well as sulfasalazine [97,98,99].

There is scarce data on the use of antibiotics in the treatment of patients with ankylosing spondylitis and psoriatic arthritis. Preliminary data suggest that antibiotics may influence disease activity by altering the gut microbiota.

### 4.5. Rheumatoid Arthritis (RA)

Treatment options for patients with RA include traditional disease-modifying anti-rheumatic drugs (DMARDs), such as methotrexate, sulfasalazine, leflunomide, and hydroxychloroquine, and corticosteroids [100]. Also, biologics, such as etanercept (a TNF inhibitor) and monoclonal antibodies (infliximab, rituximab, adalimumab, and tocilizumab), and targeted synthetic DMARDs (tsDMARDs) such as tofacitinib, baricitinib, and upadacitinib have been used [101,102,103,104,105].

Regarding the antibiotics, minocycline, doxycycline, clarithromycin, roxithromycin, and levofloxacin have been studied in small clinical trials of patients with RA [15]. In most of these trials, antibiotics led to clinical [reduction in symptoms as well as signs (joint swelling and tenderness)] and laboratory improvement [reduction in erythrocyte sedimentation rate (ESR)] [15,63,106]. In addition, a slower progression of joint damage was observed compared to the placebo. The baseline inflammation, duration, and serological status of RA influence the effectiveness of minocycline. The observed improvement may relate to the ability of certain antibiotics, particularly tetracyclines and macrolides, to attenuate synovial inflammation by down-regulating pro-inflammatory cytokines such as TNF-α and IL-6 and by inhibiting matrix-degrading enzymes. These effects could reduce local tissue destruction and systemic inflammatory activity, thereby explaining the parallel clinical and laboratory improvement seen in the trials.

A critical evaluation of the available data suggests that antibiotic therapy is not generally recommended for RA. It may be considered in early, mild, or treatment-resistant cases of RA, mainly where other recommended therapies have led to considerable adverse events.

### 4.6. Systemic Lupus Erythematosus (SLE)

Antibiotics are used to control active infection in a subset of patients with SLE. However, they are not considered standard treatment because they lack supportive data. Macrolides have been studied in animal models of lupus for their immunomodulatory effects. Preliminary data suggested a potential for reducing kidney inflammation and proteinuria [107].

Some clinicians explore macrolides or tetracyclines as adjuncts, but these are not part of routine management. More robust clinical trials are necessary to validate any immunomodulatory role in SLE [108].

### 4.7. Granulomatosis with Polyangiitis

The effectiveness of trimethoprim-sulfamethoxazole in the prevention of relapses of granulomatosis with polyangiitis was studied in a randomized, placebo-controlled trial with a 2-year duration. Specifically, the trial was conducted in 81 patients with granulomatosis with polyangiitis who were in remission. A higher proportion of patients on trimethoprim-sulfamethoxazole compared to placebo remained in remission (82% vs. 60%) [109]. In addition, there were fewer infections of the respiratory tract in patients who received the antibiotic [109].

One of the potential mechanisms of the activity of trimethoprim-sulfamethoxazole in patients with granulomatosis with polyangiitis is its effect on the microbial flora, especially of the nasal cavity. A substantial amount of data suggests the pathogenic role of alterations in the nasal microbial flora, with predominance of *Staphylococcus aureus* in the area in patients with granulomatosis with polyangiitis [110,111,112]. The persistent nasal colonization by this bacterium has been associated with increased probability of relapse. Subsequently, it is postulated that trimethoprim-sulfamethoxazole may reduce the relapse of the disease via the antibacterial effect on *S. aureus* of the nasal cavity.

### 4.8. Other Rheumatologic Diseases

In cases of arthritis, the differential diagnosis is broad. It includes local infections (septic arthritis), substance deposition arthritis (such as gout), specific rheumatologic diseases (such as RA and SLE), as well as immune complex-mediated arthritis (as in the case of infective endocarditis) [113,114]. Also, viruses have been linked to various rheumatologic manifestations such as cryoglobulinemia associated with hepatitis C virus (HCV) and polyarteritis nodosa associated with hepatitis B virus (HBV) [115,116,117,118,119].

Q-fever is a systemic zoonotic infection caused by *Coxiella burnetii*. Its clinical manifestations may include various forms of vasculitis, such as large-vessel vasculitis, leukocytoclastic vasculitis, and antineutrophil cytoplasmic antibody (ANCA)-associated vasculitis [120,121]. The infection is usually treated with a prolonged course of doxycycline [122].

## 5. Limitations of Use

The use of antibiotics is indicated for certain rheumatologic diseases with a clear infectious etiology, as described above. Their use in other rheumatologic diseases is controversial and lacks support from randomized controlled trials. The critical evaluation of the literature suggests that further research is justified on the potential role of antibiotics for patients with various rheumatologic diseases. However, the available data primarily come from basic science studies examining the possible mechanisms of antibiotic activity in patients with such diseases. In contrast, the available clinical studies are limited and have significant methodological issues, including flaws in study design, inadequate sample size, and potential bias. Subsequently, clinicians should follow the relevant guidelines and avoid overuse of antibiotics in this patient population.

On the other hand, although supporting clinical evidence remains limited, the available experimental data clearly indicate that certain antibiotic classes possess immunomodulatory properties that can influence inflammatory pathways relevant to disease activity. These preclinical findings highlight the biological plausibility of benefit and merit further investigation rather than dismissal, given current gaps in clinical evidence.

Still, the use of antibiotics for rheumatologic diseases raises concerns and poses questions related to various issues. First, antibiotics contribute to the evolving and growing antimicrobial resistance, one of the most critical, current, global public health problems [123,124]. The overuse of carbapenems, including meropenem and imipenem, has led to the development of resistance in Gram-negative bacteria to these antibiotics, including the production of *Klebsiella pneumoniae* carbapenemase (KPC) [125]. Similarly, clinical isolates of *Acinetobacter* spp. have shown an increasing prevalence of resistance due to the production of metallo-β-lactamases (MBLs) [126].

Second, they may have considerable adverse events, such as nephrotoxicity, hepatotoxicity, allergic reactions, gastrointestinal toxicity, bone marrow suppression of blood cell production, and other toxicity, especially with prolonged use [127]. Nephrotoxicity is a well-known and frequently encountered adverse reaction associated with the use of antibiotics and presents a significant concern in pharmacovigilance, while new antibiotics are constantly assessed for their nephrotoxicity potential [128]. It has been reported that increased serum β-lactam levels are associated with toxicity. This toxicity can increase morbidity and lead to chronic disability or death [27]. Third, they have a deleterious effect on the normal microbial flora, which is considered a significant concern, especially given the evolving data on the pathogenetic role of dysbiosis in the initiation and progression of common rheumatologic diseases, including SLE, RA, and juvenile idiopathic arthritis [129,130,131,132,133,134].

## 6. Future Directions

Current research in rheumatology is increasingly focused on the therapeutic potential of newly discovered molecular and cellular pathophysiologic pathways. Advances in our understanding of the underlying mechanisms of rheumatologic diseases have uncovered potential targets that offer opportunities for innovative treatments. Each step of these pathways improves our understanding of disease progression and presents therapeutic implications, from early-stage interventions to precise and personalized treatments. These findings have the potential to expand current therapeutic strategies, thereby improving both the efficacy and safety of treatments for patients with rheumatic diseases. Furthermore, the growing availability of molecular and genetic testing is supporting the advancement of personalized medicine.

Further evidence for the potential effectiveness of antibiotics in treating patients with clearly defined rheumatologic diseases should come from well-designed and adequately powered randomized clinical trials and longitudinal studies. Additionally, further research on the effects of probiotics, prebiotics, and antibiotics on the human microbiota, particularly in the gut and related mechanisms, is warranted. In addition, the discovery of antimicrobial substances with limited antimicrobial activity and essential immunomodulatory actions will be a real contribution in the field of Rheumatology. Notably, several compounds derived from antibiotics—such as chemically modified tetracyclines (CMTs) and non-antimicrobial macrolide derivatives—are being developed specifically for their immunomodulatory and anti-inflammatory actions, with promising results in preclinical models of inflammation and tissue injury [135,136,137]. Finally, modern applications of precision medicine may help predict the beneficial effects of antibiotics in patients with rheumatologic diseases by utilizing relevant genetic and microbiome markers.

Future clinical research should focus on well-designed, randomized controlled trials targeting well-defined subgroups of patients, such as those with early or treatment-refractory disease. Incorporation of biomarkers of inflammation (e.g., TNF-α, IL-6, MMPs) and host–microbiome interactions could help stratify patient response and identify potential predictors of benefit. Parallel mechanistic studies examining cytokine modulation, immune-cell signaling, and genetic susceptibility are needed to clarify the pathways through which antibiotic-derived compounds exert immunomodulatory effects.

## 7. Conclusions

A critical evaluation of the relevant published data on the role of antibiotics in rheumatologic diseases suggests that they can be used only in treating patients with specific diseases with a clear infectious etiology (Table 3). Antimicrobial agents can be used to treat and prevent the recurrence of rheumatic fever, treat Whipple’s disease, and treat early Lyme disease. Additionally, they may have beneficial effects when administered early in patients with reactive arthritis. Also, antibiotic treatment is essential for patients with Q-fever-related vasculitis.

Beyond their established antimicrobial indications, antibiotics demonstrate immunomodulatory effects in experimental and early clinical settings. While current data are insufficient to support their routine therapeutic use in non-infectious rheumatologic diseases, this absence of conclusive evidence should not be interpreted as evidence of absence. Instead, it underscores the need for well-designed studies to clarify which antibiotic-derived compounds or analogs might safely modulate inflammation for clinical benefit.

## Figures and Tables

**Table 1:** Basic pathogenetic mechanisms of infections leading to rheumatologic diseases.

Mechanism	Description	Examples
**Direct site infection**	Infection of the joint with local growth of the pathogen	Septic arthritis, tuberculous arthritis, *Brucella* arthritis
**Molecular mimicry**	Shared antigenic epitopes between microbes and the host lead to cross-reactivity, which triggers an autoimmune response against self-tissues	Rheumatic fever, SLE, reactive arthritis, ankylosing spondylitis
**Epitope spreading**	The immune response directed against a microbial agent may expand to include self-antigens released during tissue damage	SLE, RA, Sjögren’s disease, idiopathic inflammatory myopathies (dermatomyositis, polymyositis)
**Bystander activation**	Inflammatory cytokines released during infection may activate autoreactive lymphocytes that would otherwise remain quiescent	RA, SLE, Sjögren’s disease, juvenile idiopathic arthritis, ankylosing spondylitis
**Chronic active infection**	Chronic active infection leads to chronic inflammation and autoimmunity	Lyme disease, reactive arthritis, cryoglobulinemia-associated vasculitis, and arthritis with HCV

**Abbreviations**: HCV: hepatitis C virus, RA: rheumatoid arthritis, SLE: systemic lupus erythematosus.

**Table 2:** Main mechanisms of immunomodulatory effects of antibiotics considered in the treatment of patients with rheumatologic diseases.

Antibiotic	Main Mechanisms of Immunomodulatory Effects
**Macrolides**	Reduction in pro-inflammatory cytokines (IL-1β, IL-6, and TNF-α)
	Stabilization of lysosomal membranes
	Suppression of the oxidative burst
	Reduction in neutrophil chemotaxis and adhesion
**Tetracyclines**	Inhibition of matrix metalloproteinases
	Reduction in T-lymphocyte activation and proliferation
	Reduction in pro-inflammatory cytokines
	Inhibition of the activation of macrophages and microglial cells
**Fluoroquinolones**	Reduction in pro-inflammatory cytokines
	Inhibition of the activation of NF-κB
	Reduction in neutrophil chemotaxis and adhesion
	Reduction in antibody production and T-cell proliferation
	Induction of apoptosis in immune cells
**Sulfapyridine** **(in sulfasalazine)**	Interference with the function of B-lymphocytes
	Reduction in pro-inflammatory cytokines
	Inhibition of the activation of NF-κB

**Abbreviations:** IL-1β: interleukin-1β, IL-6: interleukin-6, TNF-α: tumor necrosis factor-α, NF-κΒ: nuclear factor-κΒ.

**Table 3:** Implicated bacteria as etiologic agents and recommended antibiotic treatment in specific rheumatologic diseases.

Disease	Implicated Microorganism	Clinical Manifestations	Recommended First-Line Antibiotic Treatment
**Rheumatic fever**	*Streptococcus pyogenes*	Arthritis, carditis, skin rash, subcutaneous nodules, chorea	Penicillin for the initial treatment and prevention of recurrence
**Whipple’s disease**	*Tropheryma whipplei*	Polyarthritis, diarrhea, weight loss, central nervous system involvement	Initially, intravenous ceftriaxone for two weeks, then trimethoprim-sulfamethoxazole for one year *
**Lyme arthritis**	*Borrelia burgdorferi*	Usually, involvement of one or a few joints in late Lyme arthritis	Doxycycline or cefuroxime per os; intravenous ceftriaxone in severe cases
**Reactive arthritis**	*Chlamydia trachomatis* and gastrointestinal pathogens	Usually, asymmetric oligoarthritis	Doxycycline or azithromycin for *Chlamydia trachomatis* **
**Q fever vasculitis**	*Coxiella burnetii*	Should be considered in cases of culture-negative endocarditis	Long-term doxycycline (and hydroxychloroquine)

* Long-term antibiotic treatment is recommended to decrease the probability of relapse of Whipple’s disease. ** In patients with reactive arthritis, inflammation of the joints may persist even after the eradication of implicated bacteria. The benefit of antimicrobial treatment is limited, if any, in cases of reactive arthritis with established arthritis.

## Data Availability

No new data were created or analyzed in this study. Data sharing is not applicable to this article.

## Abbreviations

ANCA	antineutrophil cytoplasmic antibody
CMTs	chemically modified tetracyclines
DMARDs	disease-modifying anti-rheumatic drugs
ESR	erythrocyte sedimentation rate
GPA	granulomatosis with polyangiitis
HBV	hepatitis B virus
HCV	hepatitis C virus
IBD	inflammatory bowel disease
KPC	*Klebsiella pneumoniae* carbapenemase
MBL	metallo-β-lactamases
MMP	matrix metallo-proteinase
MRSA	methicillin-resistant *Staphylococcus aureus*
NF-κB	nuclear factor-kappa B
PAMPs	pathogen-associated molecular patterns
PAS	periodic acid-Schiff
PSC	primary sclerosing cholangitis
RA	rheumatoid arthritis
SLE	systemic lupus erythematosus
TLRs	Toll-like receptors
